# Evaluating changes in the color and luminosity of dental enamel after orthodontic treatment: A clinical study

**DOI:** 10.1590/0103-6440202204913

**Published:** 2022-12-05

**Authors:** Lucineide Lima dos Santos, Sandrine Bittencourt Berger, Thais Maria Freire Fernandes, Flaviana Alves Dias, Murilo Baena Lopes, Paulo Henrique Perlatti D’Alpino, Alcides Gonini-Júnior, Ricardo Danil Guiraldo

**Affiliations:** 1 Department of Restorative Dentistry, School of Dentistry, University Pitagoras Unopar, Londrina, Paraná, Brazil.; 2 Biotechnology and Innovation in Health Program, University UNIAN, São Paulo, SP, Brazil.; 3 Department of Restorative Dentistry, School of Dentistry, State University of Londrina, Londrina, Paraná, Brazil.

**Keywords:** Orthodontic brackets, white spot lesions, dental cements, fluoride

## Abstract

The aims of this clinical study were to evaluate the Color change - *ΔE* (based on spectrophotometry and visual analysis) and luminosity - L* (based on spectrophotometry) of dental enamel surface (after orthodontic treatment) around the area where orthodontic brackets were fixed, based on different cementing materials such as a resin (R group) and resin-modified glass ionomer cement (RMGIC group). The split-mouth study initially comprised 14 patients. Orthodontic brackets were fixed to the upper central incisors with resin or RMGIC. The color of the buccal surface of each tooth was measured through spectrophotometry and visual examination before the bracket-fixation process. Four individuals were excluded during the follow-up; thus 10 patients were evaluated (n=10). Brackets were removed after 12 months of orthodontic treatment, tooth color measurement and visual examination were performed again, and Adhesive Remaining Index (ARI) was also measured. *ΔE* and L* results were subjected to Student's t-test and by repeated-measures analysis of variance, respectively (α=0.05). ARI data were analyzed in percentages. There was statistically significant difference in *ΔE* between groups; the R group showed statistically higher values of L* after orthodontic treatment. ARI of 2 and 3 prevailed in the RMGIC group, whereas the R group presented 0 and 1. After orthodontic treatment, the RMGIC group presented smaller changes in *ΔE,* and the increase in the white scale was observed on the enamel surface around the area where brackets were fixed in the R group. The visual analysis did not show color change on the evaluated teeth.

## Resumo

Os objetivos deste estudo clínico foram avaliar a alteração de cor - *ΔE* (baseada em espectrofotometria e análise visual) e luminosidade - L* (baseada em espectrofotometria) da superfície do esmalte dentário (após tratamento ortodôntico) ao redor da área onde os braquetes ortodônticos foram fixados, com base em diferentes materiais de cimentação, como resina (grupo R) e cimento de ionômero de vidro modificado por resina (grupo RMGIC). Estudo boca dividida foi inicialmente composto por 14 pacientes. Os braquetes ortodônticos foram fixados nos incisivos centrais superiores com resina ou RMGIC. A cor da superfície vestibular de cada dente foi mensurada por espectrofotometria e avaliada por exame visual antes do processo de fixação dos braquetes. Quatro indivíduos foram excluídos durante o acompanhamento; assim, 10 pacientes foram avaliados (n=10). Os braquetes foram removidos após 12 meses de tratamento ortodôntico, a mensuração da cor do dente e o exame visual foram realizados novamente, e o Índice de Remanescente do Adesivo (IRA) também foi mensurado. Os resultados *ΔE* e L* foram submetidos ao teste t de Student e à análise de variância para medidas repetidas, respectivamente (α=0.05). Os dados do ARI foram analisados em porcentagens. Houve diferença estatisticamente significativa no *ΔE* entre os grupos; o grupo R apresentou valores estatisticamente maiores de L* após o tratamento ortodôntico. O IRA 2 e 3 prevaleceu no grupo RMGIC, enquanto o grupo R apresentou 0 e 1. Após o tratamento ortodôntico, o grupo RMGIC apresentou menores alterações no ΔE e o aumento da escala branca foi observado na superfície do esmalte ao redor da área onde os braquetes foram fixados no grupo R. A análise visual não mostrou mudança de cor nos dentes avaliados.

## Introduction

Intact enamel is a component of a beautiful smile and orthodontic treatment is not supposed to jeopardize its original quality ^1^. There is a well-established relationship between orthodontic fixed appliance use and enamel demineralization ^2^. White spot lesions (WSL) are the initial manifestations of carious processes; they persist as one of the side effects of orthodontic treatments. The use of fixed brackets increases patients’ risk of developing caries on the buccal surface of their teeth ^3^. The main reason lies on biofilm accumulation around brackets and wires, as well as between brackets and the gingival margin. Their typical whitish and opaque appearance can be physically explained by higher light refraction in the body of the lesion than on the surrounding healthy enamel due to differences between means of transmission, such as retained air and saliva in the body of the lesion ^4^. WSL affects almost 50% of patients undergoing orthodontic treatment for 12 months ^5^. Such high prevalence is partly attributed to uneven brackets surface and to the presence of orthodontic wires, bands and other accessories that enable plaque retention, hinder oral hygiene and limit the tooth’s ability to clean itself through salivary flow and oral muscle movements ^6^. Several preventive strategies have been employed to avoid the initiation, to arrest or reverse the progression, or to mask the WSL ^7^.

Fluoride is the substance most often used in remineralization processes ^8^. The scientific basis for such use lies on the fact that fluoride ions can penetrate the crystalline structure of dental hard tissues, decrease their solubility and provide acid resistance. Fluoride ions replace hydroxyl groups in hydroxyapatite formulation and lead to fluorapatite formation ^3^. The use of fluoride-releasing materials to bond orthodontic accessories could help minimize and prevent the emergence of these injuries. Glass ionomer stands out among these materials since it is capable of chemically adhering to the enamel and releasing fluorine, however, exhibit lower mechanical properties to resin materials ^9^. The evolution of these types of cement and the incorporation of resin matrix to their formulation have improved their adhesion to orthodontic brackets ^10^, which is the reason why they are indicated for this purpose.

Direct visual examination is the method most often used to detect WSLs. Although it is a good approach, it is hard for a single investigator to consistently assess a large number of patients in different clinical settings or at different periods of time ^3^. Thus, the aims of this clinical study were to evaluate the Color change - ΔE (based on spectrophotometry and visual analysis) and luminosity - L* (based on spectrophotometry) of dental enamel surface (after orthodontic treatment) around the area where orthodontic brackets were fixed, based on different cementing materials such as resin (R group) and resin-modified glass ionomer cement (RMGIC group). The herein tested hypotheses were that the different cementing materials used in the current study can influence changes ^1^ in the color and ^2^ luminosity of the dental enamel surface around the area brackets were fixed after orthodontic treatment.

## Material and Methods

### Ethical Considerations

The present project was a clinical study focused on evaluating changes in the color of tooth enamel surface based on spectrophotometry assessments used to measure tooth color before brackets fixing by using different cementing materials and after brackets removal. It was submitted to UNOPAR ethics and research committee and approved under number 2,931,538.

### Sampling Estimation

Sample size was calculated based on the standard deviation of 1.7 primary results recorded for color (*ΔE*) in a previous study ^11^ by taking into consideration the minimum detectable difference of 3.0 in the mean. A total of 8 participants were required (Variance Test, not-equal, 95% of CI, power of 80%).

The study followed the split-mouth approach conducted with fourteen patients who needed orthodontic treatment. The patient selection was based on the following inclusion criteria: individuals presenting a high risk of developing caries, who were assessed based on the visible plaque index - wherein 1 was classified as visible plaque and 0 was classified as absence of plaque ^12^; whose upper central incisors were free of caries, enamel defects, restorations, history of trauma or changes in salivary flow. Patients whose upper central incisors presented fractures, composite resin restorations, cracks or enamel hypoplasia, and who had already used orthodontic appliance, were excluded from the study.

**Figure 1:**
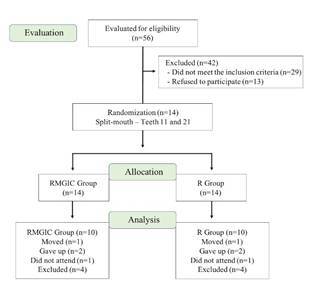
Flowchart of patients. RMGIC: resin-modified glass ionomer cement. R: resin.

The flow of participants (Figure 1): 14 patients (8 boys and 6 girls) in the age group 12-17 years were selected for the split-mouth study and had each of their upper central incisors allocated to two different experimental groups: 14 in the RMGIC group and 14 in the R group. Sample recruitment was carried out from May to July 2018. Four individuals were excluded during the follow-up: 1 of them moved to another city, 2 patients abandoned treatment and 1 patient did not attend the final color assessment; thus, the final sample comprised 10 participants (5 boys and 5 girls) in each group (n=10).

### Clinical procedures

The buccal surface of each incisor was subjected to prophylaxis with pumice paste (SS White, Petrópolis, RJ, Brazil) and water with the aid of a rubber cup at low rotation (Dabi-Atlante, Ribeirão Preto, SP, Brazil) for 10 seconds. A spectrophotometer (EasyShade Advance 4.0; VITA, Zahnfabrik, Germany) was used to measure the color of the buccal face of the incisors (11 and 21) after prophylaxis.

Premium Series metallic brackets (Orthometric, Marília, SP, Brazil) with MBT prescription, slot 0.022”x0.028", were randomly selected on the surface of the upper central incisors based on cementing materials: resin-modified glass ionomer group (Vitremer; 3M ESPE, St. Paul, MN, USA) and resin group (Transbond XT; 3M Unitek, Monrovia, CA, USA).

Both cements were applied at the basis of the brackets and positioned, with the aid of orthodontic tweezers (Morelli, Sorocaba, SP, Brazil), on the enamel surface, which was subjected to manual pressure to overflow the excess of cement that was removed with micro brush (FGM, Joinville, SC, Brazil). The direct brackets fixing technique, based on the use of Transbond XT and Vitremer resin, was applied to teeth, according to instructions by the manufacturers.

The brackets were removed after 12 months of orthodontic treatment. The vestibular surface of the upper central incisors was subjected to finishing and polishing procedures at low rotation, with the aid of a handpiece equipped with aluminum oxide discs (Sof-lex; 3M ESPE) for 20 s each to both groups, which were used at decreasing abrasiveness order to remove the remaining adhesive. Thus, the polishing time and abrasiveness of the discs were standardized according to a previous study ^13^. The orthodontic treatment was planned based on the needs of each patient participating in the study; all patients were instructed to use mouthwash ^6^ composed of 10 ml of 0.2% sodium fluoride solution made by the same manufacturer (Flora Farmácia de Manipulação, Salvador, BA, Brazil) for one minute, once a week. They received oral hygiene instructions at the beginning of the treatment and at each follow-up visit. There was no brackets detachment from units 11 and 21 of any patient treated in the current study.

### Spectrophotometry Analysis

The spectrophotometer (EasyShade) was used to measure the color on the vestibular surface of units 11 and 21 in the cervical third (middle, mesial and distal) of the buccal surface. Three measurements were taken, and the mean of these measurements was calculated. Color measurements were performed before, and 12 months after, the orthodontic treatment.

Color change (*ΔE*) was evaluated by comparing the initial and final measurements of each tooth, based on parameters set by Commission International de l'Éclairage (CIE) to evaluate L*, a* and b* values. According to this method, color is three-dimensionally analyzed by taking into consideration the following coordinates:

L*: luminosity, which is determined based onblack-and-white variations in luminosity, at different times;

A*: it determines variations between the amount of red (positive values) and green (negative values) color, at different times;

b*: it determines variations between the amount of yellow (positive values) and blue (negative values) color, at different times.

Color variation (ΔE) was determined based on the Hunter equation:

$${\Delta E}^{*}=\;{\left[{\left({\Delta L}^{*}\right)}^{2}+{\left({\Delta a}^{*}\right)}^{2}\;+\;{\left({\Delta b}^{*}\right)}^{2}\right]}^{1/2}$$

Comparisons between the emergence of whitespotst in units 11 and 21 were based on parameter L*, by taking into consideration the color change detected by the spectrophotometer, in association with the adopted brackets fixing material. Thus, L*i (L* initial - before orthodontic treatment) and L*f (L* final - after orthodontic treatment) were used as a parameter.

### Adhesive Remnant Index Analysis

The Adhesive Remnant Index (ARI) was analyzed before polishing was carried out. The vestibular surfaces of the teeth were evaluated by two examiners (neither of whomwas involved in the bonding procedure), in a blind analysis. ARI was used to classify the failure mode as follows: 0, absence of any residue from the adhesive layer on the enamel; 1, presence of less than half of the remaining resin on the enamel; 2, presence of more than half of the remaining resin on the enamel; and 3, presence of all remaining resin on the enamel, together with the impression left by the brackets on its basis ^14^. This evaluation was performed twice by the same operator, at a 30-minute interval between evaluations

### Visual Analysis

The upper central incisors were isolated with cotton rolls and air-dried for 5 seconds before measurements. The cervical surface of the tooth (middle, mesial and distal) was evaluated to investigate the presence of WSL ^5^ since this area is the most prone to present enamel demineralization during orthodontic treatments.

### Blinding

The initial and final evaluations of color measurements and the visual analysis were performed blindly, based on a double-blind study, in which the evaluator and the patient were blind.

### Statistical Analysis

Color change (*ΔE*) and luminosity (L* coordinate) results were subjected to the Kolmogorov-Smirnov normality test, which was followed by Student's t-test for ΔE analysis and by repeated-measures analysis of variance for L* coordinate evaluation, at 5% significance level. A*i (initial - before orthodontic treatment), A*f (final - after orthodontic treatment), b*i (initial - before orthodontic treatment), b*f (final - after orthodontic treatment) coordinates were subjected to Student's t-test for different groups. ARI data were analyzed in percentages.

## Results

Color change magnitude was assessed based on *ΔE*; this parameter presented statistically significant difference (p=0.0114) between groups (Table 1). The cervical surface of the tooth (middle, mesial and distal) did not show the presence of WSL.

**Table 1:** Mean color change (*ΔE*) for different groups.

Group	*∆E*	95% CI (Lower limit / Upper limit)	*p*
RMGIC	3.164 (1.164) B	2.232 / 4.096	0.0114
R	4.866 (1.607) A	3.93 / -5.798	

Mean values followed by different uppercase letters in rows and lowercase letters in columns are significantly (Student's t-test). RMGIC: resin-modified glass ionomer cement. R: resin. CI: Confidence Interval. Standard deviations are in parentheses

Luminosity measured based on parameter L* did not change between the initial and final moments in the RMGIC group (p=0.68) and in the comparison of initial moments between groups (p=0.33). However, luminosity measured at the final moment has shown a significant difference between groups (p <0.001) and between the initial and final moments in the R Group (Table 2).

**Table 2:** Mean of the L*i (L* initial - before orthodontic treatment) and L*f (L* final - after orthodontic treatment) and *ΔL* for different groups.

Group	L*i	L*f	*p*	*ΔL*
RMGIC	82.91 (0.95)	82.78 (0.46)	0.68	-0.13
R	82.5 (0.68)	84.01 (0.44)	<0.001*	1.43
*p*	0.33	<0.001*		

*Significantly (repeated-measures analysis of variance). RMGIC: resin-modified glass ionomer cement. R: resin. Standard deviations are in parentheses.

The A*i, A*f, b*i and b*f coordinates did not show statistical difference between the different groups (p=0.8837, p=0.6083, p=0.602, p=0.2284; respectively).

Results recorded for ARI scores are shown in Table 3. Scores 2 and 3 and scores 0 and 1 prevailed in the RMGIC and R groups, respectively.

**Table 3:** Frequency distributions of adhesive remnant index (ARI) scores.

Group	0	1	2	3
RMGIC	0	20.0	40.0	40.0
R	30.0	40.0	10.0	20.0

RMGIC: resin-modified glass ionomer cement. R: resin. The ARI was used to classify the failure mode as follows: 0, no bonding resin left on the tooth; 1, less than half of the bonding resin left on the tooth; 2, more than half of the bonding resin left on the tooth; and 3, all bonding resin left on the tooth, with distinct impression of the bracket mesh.

## Discussion

The emergence of white spots - incipient lesions - on the enamel is one of the most common and anti-aesthetic effects of orthodontic treatment with fixed brackets. The use of bonding materials capable of releasing fluorides would be a way to helpprevent these injuries. Thus, the aims of this clinical study were to evaluate the Color change - ΔE (based on spectrophotometry and visual analysis) and luminosity - L* (based on spectrophotometry) of dental enamel surface (after orthodontic treatment) around the area where orthodontic brackets were fixed, based on different cementing materials such as a resin (R group) and resin-modified glass ionomer cement (RMGIC group). A split-mouth study ^15^ has eliminated individual differences such as saliva pH, oral hygiene and diet to allow each patient to be their own control; results have shown possible cross-contamination from one side to the other, which was the limitation of the aforementioned study. In addition, the generalization of these results is limited to patients whose brackets were fixed with RMGIC or resin - based on recommendations by the manufacturer about materials used in the present study, which may undergo changes due to different bonding techniques. A study ^16^ found no difference in the bracket failure rate when brackets were bonded using RMGIC or resin. Similarly, a single arm randomized controlled trial ^17^ demonstrated a clinically acceptable bracket failure rate with RMGIC.

Objective color analysis was performed in a spectrophotometer to provide accurate quantitative data ^(^ ^18^. Color change values were evaluated based on reflectance measurements carried out in CIE Lab color coordinate system. When color dimension coordinates L*, a* and b* were analyzed in separate, L* values, which represent the luminosity of the object, appeared to be the most relevant parameter to be used for comparisons under experimental conditions ^19^. In addition, *ΔE* values were analyzed because they can indicate color change magnitude at two different times ^20^.

Tooth color is mainly determined by dentin, as suggested in a previous study ^21^; however, another study has shown that changes on the enamel surface can affect light dispersion or reflection ^22^. In addition, tooth color can be regulated by the size of the enamel hydroxyapatite crystals ^23^. Thus, *ΔE* evaluation was performed in the two different groups at the time to analyze changes in color magnitude at the initial and final treatment stages. The RMGIC group that contains fluoride showed statistically inferior results (p = 0.0114) of color magnitude variation when compared to the R group. This outcome suggests that fluoride release in the RMGIC group has decreased the color change promoted by factors associated with patients’ oral environment. However, the composition of RMGIC is very different from the resin, thus, future studies are needed to confirm our hypothesis.

The difference in the refractive index of the enamel crystals has contributed to the whitish nature of the WSL. These crystals present lower L* values because large portions of the transmitted light are absorbed by, and dispersed in, the micropores of the lesion body ^24^. Therefore, luminosity values are of paramount importance. A study focused on investigating artificial caries produced in tooth samples has found increased L* and varying *ΔL*, whose mean value reached 4.1826 ^24^. When the two groups (R and RMGIC) were compared, they had L*i values similar (before orthodontic treatment), with no significant difference (p=0.33). However, after orthodontic treatment, when comparing the groups, the L*f value underwent significant changes for the R group (p <0.001). Increased L* value means whiter tooth surface, which may suggest that the enamel surrounding the area where brackets were fixed to with RMGIC had better luminosity stability, since the structure of enamel crystals underwent lesser changes. However, the change (*ΔL*) measured in the spectrophotometer reached 1.43; according to a previous study ^24^, this value does not correspond to WSL. In clinical terms, although these values have undergone statistically significant changes, there were no noticeable changes capable of pointing towards the emergence of white spot. Thus, the present study has shown that the use of RMGIC to fix brackets can inhibit tooth luminosity and color changes before the emergence of visible white spots. Extremely important results in anterior teeth such as the maxillary central incisors.

The ARI scoring system is an assessment tool used to investigate adhesive systems. It is a quick and simple method that does not require special equipment ^25^. Results in the current study have shown the prevalence of scores 2 and 3 in the RMGIC group in comparison to the R group, which presented a prevalence of scores 0 and 1 (Table 4); this result corroborates the previous study ^26^, which found similar results. One hypothesis for these findings is that the use of fluorides to prevent caries promotes the incorporation of fluoride ions in the hydroxyapatite crystal network of hard tissues and leads to a mineral phase less soluble ^8^ and more resistant than hydroxyapatite.

Fixed appliances serve as plaque retention sites, and, in the absence of good oral hygiene, plaque accumulates and acidogenic bacteria cause marked demineralization ^5^. For this reason, the present study chose to use the presence of plaque as an inclusion factor. Moreover, Detecting WSLs during active treatment can be challenging for the clinician. The clinical crown must be free from plaque and debris, and the presence of excess gingival tissue can make visualization of WSLs difficult ^5^. A clinical diagnosis method based on visual inspection was carried out after samples were dried with air jet. The evaluation based on this method did not show the presence of white spotlesions in the present study for different groups, which contradicts another study ^5^, whose authors have found sharp increase in the number of white spots during the first six months of treatment, which continued to slowly increase until the 12^th^ month. However, some advantages of using RMGIC must be taken into account. In clinical terms, RMGIC use would be a better option than resin under humidity and enamel defect conditions, because it is a hydrophilic material easier to be removed, which makes this step less unpleasant for patients ^27^. The use of RMGIC in the present study promoted lower-magnitude of tooth color change than the group whose brackets were cemented with resin. Thus, hypotheses of the current study were confirmed, since there was difference between the two materials in the evaluation of color change (ΔE) and luminosity (L*) parameters. However, in the present study, only the maxillary central incisor was evaluated, which is a limitation of the study. Theoretically, this dental element would be the easiest to remove from the biofilm. Thus, other more severe results could be found and could validate the use of the RMGIC. In this way, other studies are needed to confirm this finding.

Another limitation of the study was n=10, considered small for a clinical study. However, the present study started with 56 patients (Figure 1), 42 were excluded (29 due to not meeting the inclusion criteria and 13 due to refuse to participate). Thus, 14 patients were restored to the allocation, of which 4 were excluded from the analysis (1 moved, 2 gave up, 3 did not attend). This loss of patients was a challenge found in the present study, however, there is a clinical study with the same number of patients ^28^. Thus, this fact is a limitation of the present study that does not prevent its publication. Therefore, based on this clinical study, the following conclusions can be drawn: Spectrophotometry measurements have shown color change on the enamel surface around the area brackets were fixed to with resin, after orthodontic treatment; Visual analysis did not show color change on the evaluated teeth; Increased white scale was observed on the enamel surface around the area where brackets were fixed to with resin, after orthodontic treatment.
